# Immune Functions of Astrocytes in Viral Neuroinfections

**DOI:** 10.3390/ijms24043514

**Published:** 2023-02-09

**Authors:** Jernej Jorgačevski, Maja Potokar

**Affiliations:** 1 Laboratory of Neuroendocrinology–Molecular Cell Physiology, Institute of Pathophysiology, Faculty of Medicine, University of Ljubljana, Zaloška 4, 1000 Ljubljana, Slovenia; 2 Celica Biomedical, Tehnološki Park 24, 1000 Ljubljana, Slovenia

**Keywords:** central nervous system, astrocyte, neuroinfection, virus, immune response, pattern recognition receptor, cytokine

## Abstract

Neuroinfections of the central nervous system (CNS) can be triggered by various pathogens. Viruses are the most widespread and have the potential to induce long-term neurologic symptoms with potentially lethal outcomes. In addition to directly affecting their host cells and inducing immediate changes in a plethora of cellular processes, viral infections of the CNS also trigger an intense immune response. Regulation of the innate immune response in the CNS depends not only on microglia, which are fundamental immune cells of the CNS, but also on astrocytes. These cells align blood vessels and ventricle cavities, and consequently, they are one of the first cell types to become infected after the virus breaches the CNS. Moreover, astrocytes are increasingly recognized as a potential viral reservoir in the CNS; therefore, the immune response initiated by the presence of intracellular virus particles may have a profound effect on cellular and tissue physiology and morphology. These changes should be addressed in terms of persisting infections because they may contribute to recurring neurologic sequelae. To date, infections of astrocytes with different viruses originating from genetically distinct families, including Flaviviridae, Coronaviridae, Retroviridae, Togaviridae, Paramyxoviridae, Picomaviridae, Rhabdoviridae, and Herpesviridae, have been confirmed. Astrocytes express a plethora of receptors that detect viral particles and trigger signaling cascades, leading to an innate immune response. In this review, we summarize the current knowledge on virus receptors that initiate the release of inflammatory cytokines from astrocytes and depict the involvement of astrocytes in immune functions of the CNS.

## 1. Introduction

**Figure 1:**
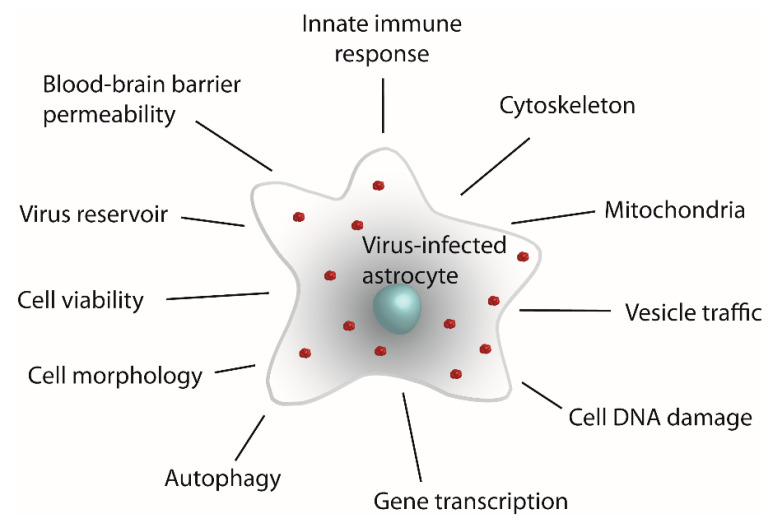
Viral infection of astrocytes affects a set of cellular processes that alter the morphologic and physiologic properties of these cells. The schematic depicts an astrocyte infected with virus (red clusters) and cellular entities and processes that undergo changes triggered by viral infection.

Astrocytes are glial cells with key roles in maintaining the homeostasis of the central nervous system (CNS) [1,2,3]. In addition to their well-recognized roles in sustaining and modulating functioning of neurons in normal physiologic conditions, they also participate in the development and progression of several diseases of the CNS [2,3,4,5]. An increasing amount of data on viral infections of astrocytes, in conjunction with further knowledge of cellular functions that are modified by virus infections (e.g., upregulation of cytokines, vesicular traffic, and autophagy; Figure 1) [6,7,8,9,10,11], have led to a whole new perspective on astrocytes in terms of their contribution to CNS diseases. To date, infections of astrocytes have been documented for viruses from different families, including enveloped positive-sense single-stranded RNA viruses (e.g., Flaviviridae, Coronaviridae, Retroviridae, and Togaviridae), enveloped negative-sense single-stranded RNA (e.g., Paramyxoviridae, Rhabdoviridae, and Bunyaviridae), non-enveloped viruses with a single-stranded RNA (Picomaviridae), and enveloped double-stranded DNA viruses (Herpeseviridae).

A confirmed infection and/or the decisive role of astrocytes in the replication of various viruses, compared with other types of brain cells and endothelial cells that constitute the blood–brain barrier (BBB), have been demonstrated so far for flaviviruses, e.g., tick-borne encephalitis virus (TBEV), Japanese encephalitis virus (JEV), Zika virus (ZIKV), West Nile virus (WNV), and Kyasanur Forest disease virus (KFDV) [6,7,12,13,14,15,16,17]; retroviruses, e.g., human immunodeficiency virus-1 (HIV-1) [18]; togaviruses, e.g., western equine encephalitis virus (WEEV) [19]; paramyxoviruses, e.g., avian orthoavulavirus 1 [20], Nipah virus [21]; rhabdoviruses, e.g., rabies virus (RABV) [22]; picornaviruses, e.g., human enterovirus 71 (EV71) [23], Ljungan virus (LV) [24], Theiler’s murine encephalomyelitis virus (TMEV) [25], and vesicular stomatitis virus (VSV) [26,27]; herpesviruses, e.g., Epstein–Barr Virus (EBV) [28], human herpesvirus 6B (HH6B) [29,30]; and bunyaviruses, e.g., Crimean–Congo hemorrhagic fever virus (CCHFV) [31]. Astrocytes are also open to infection with coronaviruses, e.g., human coronavirus OC43 (HCoV-OC43), Middle East respiratory syndrome coronavirus (MERS-CoV), and severe acute respiratory syndrome coronavirus 2 (SARS-CoV-2). However, coronavirus infection does not always produce infectious virions in astrocytes [32,33,34,35,36]. Astrocytes are also susceptible to infection with La Crosse virus and mosquito-only flavivirus mosquito-borne pathogens, although infection of such cells is mostly non-productive [11,37]. The common denominator of infection with neurotropic viruses is the induction of a reactive astrogliosis [38]. Reactive astrogliosis ‘is the process whereby, in response to pathology, astrocytes engage in molecularly defined programs involving changes in transcriptional regulation, as well as biochemical, morphological, metabolic, and physiological remodeling, which ultimately result in gain of new function(s) or loss or upregulation of homeostatic ones [39,40]. These changes, in combination with an induction of innate immune response, trigger neurologic symptoms such as encephalitis, myelitis, postencephalitic parkinsonism associated with the loss of dopaminergic neurons, paralysis, and convulsions [9,19,20,41,42]. In this review, we describe the receptors by which astrocytes sense virus infection, and cytokines, which, when released from astrocytes, have the potential to trigger neurologic conditions that are often similar to certain neurologic disorders.

## 2. Immune Responses of Virus-Infected Astrocytes

### 2.1. Types of Viral Infections

Although most viruses cause acute self-limiting infections, some viruses are able to establish persistent infections by evolving sophisticated relationship with their hosts/host cells and by highjacking a wide array of cellular mechanisms for their own benefit.

During persistent infection, the virus is not cleared from the host after the primary infection [43]. Persistent infections include chronic focal infection (CFI), chronic diffuse infection (CDI), latent infection, and abortive infection. These types of infection differ in the quantity of infected cells and in the impact of the infection on cell viability. In a CFI, the virus is maintained in a small number of susceptible cells, which release the virus before their demise; in a CDI, all the cells are infected, and the virus continues to multiply without affecting cell viability [43]. In contrast to CFI and CDI, latent infection triggers episodes of recurrent disease, but between these episodes, the virus cannot be detected. Interestingly, various stimuli, such as superinfection by another virus, physical stress, or trauma, may reactivate the infection [43]. In contrast to these typical persistent infections, during abortive infection, cells do not produce any progeny virus [44].

Persistent infections can endure because of various mechanisms, e.g., non-productive infection (e.g., herpesvirus latency), proviral integration into the host genome (e.g., retroviruses), and/or continuous viral replication (e.g., flaviviruses, arenaviruses, and polyomaviruses (reviewed in [45]).Various viruses have evolved distinct mechanisms to enable permanent infections, yet they all share certain traits with high potential to trigger persistent infections, including (i) selection of cell subsets ideal for long-term maintenance of the viral genome, (ii) modulation of viral gene expression, (iii) viral subversion of cellular apoptotic pathways, and (iv) avoidance of clearance by the immune system (reviewed in [45]).

### 2.2. Viral Infections of Astrocytes

During inflammation of the CNS, astrocytes play multiple roles governed by their own intrinsic neurotoxic activities, by activation of CNS-resident microglia, and by the recruitment of peripheral inflammatory cells [46]. In general, activation of immune pathways by astrocytes is initiated after various insults, such as brain injury, ischemia, and various neuroinfections, including viral infections [8,47]. Viruses reach the CNS by various routes, including by traversing the endothelial cells of brain capillaries, penetrating through the barrier between meningeal blood and cerebrospinal fluid, and through infected peripheral and olfactory neurons [41,48,49,50]. Astrocytes are one of the earliest cell types that acquire and successfully replicate CNS-invading viruses [6,7,9,41]. These cells promptly respond to virus entry by releasing immunomodulatory molecules, thereby participating in the innate immune response, which represents the first line of defense against virus infection [51]. The contribution of astrocytes to the neurotoxic and neuroprotective roles of the innate immune response in the CNS is increasingly acknowledged.

The antiviral response of astrocytes during the early stage of viral infection acts in an opposing manner, namely, to the benefit of either the host or the virus. The role of protecting the host is mediated by the production of antiviral mediators, preventing virus replication and dissemination [8,52,53]. Conversely, astrocytes may promote replication and dissemination of viruses between cells in the CNS and act as a virus reservoir to maintain the long-term presence of the virus in the tissue [7,54].

#### Persisting Viral Infections of Astrocytes

Persisting infections in the CNS are underestimated and an underexplored aspect of viral infections. Recently, certain viruses in the CNS, including TBEV, ZIKV, HIV-1, JEV, CCHFV, and RABV, have been attributed with the potential to maintain persisting infections of astrocytes [6,7,9,18,31,52,55,56,57,58,59,60]. For example, ZIKV-infected activated astrocytes may act as a ZIKV reservoir with the ability to replicate within foci in the mouse brain for more than a year [61]. Moreover, human fetal astrocytes can support chronic ZIKV infection and continuous viral shedding for at least 1 month [15]. In the case of ZIKV, speculation has emerged that persistent ZIKV replication and consequent surrounding inflammation of the CNS with ongoing apoptosis contribute to neurologic deficits and even worsen the long-term neurologic prognosis, yet the relative contribution of persistent inflammation in the CNS parenchyma, acting to limit the spread of the virus, remains to be evaluated [61]. In addition, it has been suggested that EBV persistently infects astrocytes; however, this also has to be further evaluated together with potential consequences on the CNS [28,62].

With the progressive research on immune functions of astrocytes, it is becoming clear that these cells are engaged in acute and persistent infections of different types. However, the role of astrocytes in infections with specific viruses remains to be addressed in detail. Particularly intriguing is persistent infection of astrocytes because that may affect the functioning of the CNS in the long run. Abortive infection of astrocytes and induction of innate immune responses may play important roles in the process of infection itself and in regulating downstream adaptive immune pathways [22,63]. Abortive infection is frequently observed in the experimental design, and for example, in infection with the cervical cancer cell line HeLa and U2OS sarcoma cells with herpes simplex virus (HSV-1), the viral genome remained in a quiescent state at least for 5 weeks [44]. Moreover, indications that astrocytes can establish persistent chronic infection comes from studies on human astrocytes infected with HIV-1; rat astrocytes infected with TBEV; and the presence of CCHFV and ZIKV in astrocytes of infected mouse brain for several weeks and up to 22 weeks, respectively [6,31,61,64]. Persistent inflammation in the CNS infected with a virus can cause tissue damage due to viral tropism in the specific brain regions, and the immune responses triggered by viral infection or persistent presence of viral RNA and neurologic symptoms may last long after viral clearance [65].

**Figure 2:**
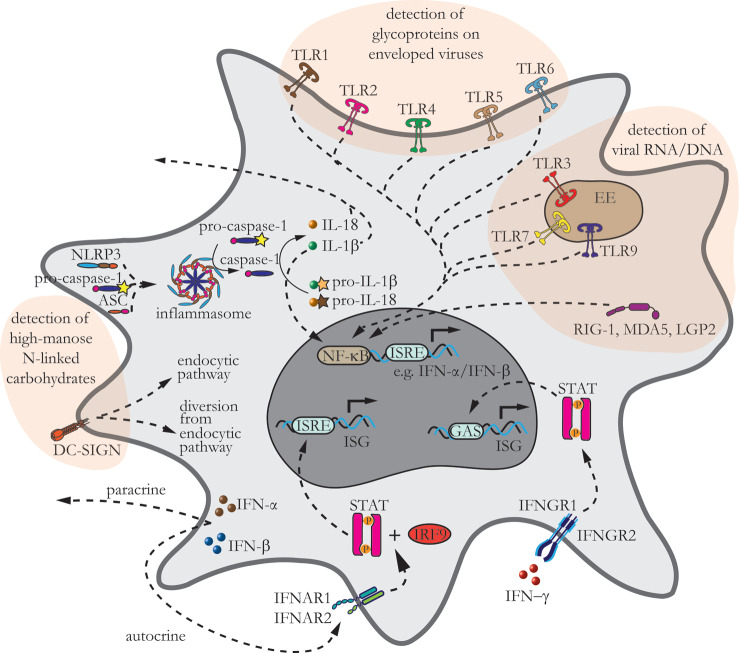
Astrocytes express several pattern recognition receptors (PRRs), which detect viral infection. The schematic depicts an astrocyte with a variety of PRRs engaged in triggering signaling cascades of the innate immune response. TLR1, TLR2, and TLRs 4-6, are located at the plasma membrane and detect glycoproteins on the viral envelope, whereas TLR3, TLR7, and TLR9 are located at the membrane of early endosomes. TLR3 detects double-stranded intermediate RNA (dsRNA), TLR7 detects single-stranded RNA (ssRNA), and TLR9 detects double-stranded DNA (dsDNA). RLRs are localized in the cytosol and sense viral RNA and DNA; they comprise RIG-I, melanoma differentiation-associated protein 5 (MDA5), and laboratory of genetics and physiology 2 (LGP2). Nucleotide oligomerization domain (NOD)-like receptors (NLRs) are another group of PRRs in the cytoplasm of astrocytes. NLRP3 contains pyrin N-terminal effector-binding domain (NLRPs), which together with pro-caspase-1 and ASC, make up the inflammasome; here, pro-inflammatory caspase-1 is converted to active caspase-1, which processes inactive interleukin (IL) precursors pro-IL-1β and pro-IL-18 to their active forms IL-1β and IL-18. IL-1β and IL-18 are either secreted from cells or activate nuclear factor-kappa B (NF-κB). CLRs are expressed at the cell surface, where they detect high-mannose N-linked carbohydrates on endogenous molecules and pathogens. Dendritic cell (DC)-specific ICAM-3 grabbing non-integrin (DC-SIGN) internalizes viruses that can either enter endocytotic pathway, ultimately resulting in viral degradation and antigen presentation, or can escape endocytic pathway. The CLR-related viral cycle in astrocytes has been poorly addressed thus far. PRR-triggered activation and translocation of IFN-regulatory factors (IRFs) and NF-κB initiate the production of inflammatory cytokines and type I interferons (IFNs), IFNα/β. IFNα/β act in paracrine and autocrine ways via interferon receptors (IFNARs) through which they initiate signaling cascades, leading to the transcription of interferon-stimulated genes (ISGs) acting through IFN-stimulated regulatory elements. IFN-γ secreted by antigen-presenting cells further assists IFNα/β in clearing virus from the tissue. IFN-γ acts through interferon-gamma receptor (IFNGR) protein complex IFNGR1 and IFNGR2, initiating a signaling cascade on gamma interferon activation site (GAS) elements in the DNA, inducing cytokine transcription.

During all types of viral infections, acute and persistent, astrocytes become engaged in immune responses, and to this end, they sense the presence of viruses through a variety of receptors which, via signaling cascades, initiate the innate immune response (Figure 2).

### 2.3. Pattern Recognition Receptors in Astrocytes

Astrocytes are now also recognized as crucial mediators of innate and adaptive immune responses in the injured CNS [66]. However, the exact mechanisms by which these cells are involved in the immune response after infection with viruses are still a topic of ongoing research. In general, primary sensing of viral infection is mediated by pattern recognition receptors (PRRs) that initiate innate immune signaling by recognition of viral particles [67]. PRRs are proteins that bind to conserved patterns present in various pathogens, and they trigger signaling events that activate innate and adaptive immunity [68]. PRRs are a versatile group of receptors: Toll-like receptors (TLRs), NOD-like receptors (NLRs), C-type lectin receptors (CLRs), retinoic acid-inducible gene I (RIG-I)-like receptors (RLRs), OAS-like receptors, and AIM-like receptors [69,70,71]. The latter two have not been described in astrocytes. Nevertheless, astrocytes modulate the innate immune response similar to professional immune cells in the CNS (microglia, monocytes, macrophages, and dendritic cells) and to other nonprofessional immune cells such as epithelial cells, endothelial cells, and fibroblasts [47,66,69,72]. PRRs that sense viral particles in astrocytes are distributed at the cell surface as well as intracellularly (Figure 2), and they recognize enveloped and non-enveloped RNA and DNA viruses through specific interactions, instigating an innate immune response (Table 1).

#### 2.3.1. Toll-like Receptors in Astrocytes

TLRs are archetypal type I transmembrane PRRs that sense various exogenous pathogens, including protozoa, bacteria, fungi, and viruses [78]. These proteins are among the first receptors to encounter viral constituents and are in general upregulated on internalization of the virus into host cells, including astrocytes [51,52,70,78]. Early immune responses conveyed from TLRs limit the replication and spread of the virus [52]. In cells that are involved in the innate immune response, different types of TLRs enable sensing of viruses from the extracellular space and in the host cell cytoplasm after the release of the internalized viral genetic material from the capsid [70]. In cells in general, TLRs 1, 2, 4, 5, and 6 are localized at the plasma membrane, and TLRs 3, 7, 8, and 9 are intracellular and likely signal from acidic endosomes [78]. All the plasma membrane TLRs recognize virus glycoproteins of enveloped viruses, and TLRs in endosomes or endolysosomes detect virus genetic material; these include TLR3, which detects double-stranded intermediate RNA (dsRNA), TLR7 and TLR8, which detect single-stranded RNA (ssRNA), and TLR9, which senses double-stranded DNA (dsDNA) [51,52,69].

Similar to other immune responsive cells, TLRs in astrocytes reside at the plasma membrane and in the membrane of intracellular compartments [52,70], but astrocytes do not express all TLRs (Figure 2). Under resting conditions, astrocytes express TLRs 2–4, and TLR2 and TLR4 may be upregulated on infection, as demonstrated with stimulation by lipopolysaccharide or treatment with poly(inosinic acid):poly(cytidylic acid) (polyI:C), which is a synthetic analog of dsRNA commonly used as a model of pathogen infections [79,80,81]. Human astrocytes predominantly express TLR3; hence, the most prominent response in these cells is to be expected especially from the activation of this particular TLR [73]. The expression of other TLRs in human astrocytes under resting conditions is much lower; for example, the expressions of TLRs 1, 4, 5, and 9 were moderate; those of TLRs 2, 6, 7, and 10 were detected faintly; and that of TLR8 was not detected at all [73]. It was recently revealed that plasma membrane TLR10, which is the only family member with poorly defined ligands, binds the HIV-1 envelope glycoprotein in breast milk cells [82]. However, despite its low expression in non-infected astrocytes, the possible role of this receptor in viral infection of astrocytes cannot be completely ruled out.

Activation of the innate immune response in the CNS is tailored according to the cell type and the environmental pathogen, and differences in the upregulation of TLRs in microglia and astrocytes have been observed [73]. In microglia, TLR4 is downregulated and TLR2 and TLR3 are subjected to positive feedback after TLR activation, but in astrocytes, all three TLRs (2–4) are upregulated after treatment with polyI:C [73].

The contribution of TLRs to neuroinfections in astrocytes has been studied increasingly in recent years, revealing that enveloped viruses are recognized by TLR2, TLR4, and TLR5 localized at the plasma membrane, and the genetic material in the cytoplasm is sensed by TLR3, TLR7, and TLR9, which reside in endosomal membranes [52]. In these studies, in addition to polyI:C treatments, TLR upregulation on actual virus infection has also been demonstrated in astrocytes, although these studies are scarce. For example, EV71-infected astrocytes upregulate TLR7 [23], and TLR3 and TLR7 have been demonstrated to participate in JEV-induced inflammatory responses in the brain [83]. The expressions of TLR3 and TLR4 were increased by infection with different ZIKV strains, and the expression of TLR5 appeared unaffected 2 days after infection [10]. Detailed expression patterns of these two and other TLRs and their role in infections with specific viruses in astrocytes are currently unknown (e.g., possible impacts on autophagy and on the release of cytokines) [10,83,84].

Despite the versatility in expression and localization patterns of different TLRs, activation of TLRs initiates common signaling cascades, which are also preserved in astrocytes (Figure 2). Generally, these cascades, which have been reviewed extensively elsewhere [69,70], lead to the activation of transcription factors that culminate in the production of cytokines. Viral sensing through TLRs present in endosomes and at the cell surface activate IRFs and NF-κB, respectively, and they jointly initiate the production of type I IFNs, IFNα/β, and inflammatory cytokines [51,69,85]. However, TLRs may have dual functions on neuroinfection. For example, the major function of TLR3 is promoting antiviral responses, but its activation can be also detrimental to the host. During infection with WNV, TLR3-deficient mice were found to be more resistant to the lethal WNV infection; the authors hypothesized that this was due to reversible breakdown of the BBB evoked by a TLR3-dependent inflammatory response [74]. In line with this view, compromised impermeability of the BBB was also demonstrated after infection with other viruses, as discussed in the Section 4.

#### 2.3.2. NOD-like Receptors in Astrocytes

NLRs are a specialized group of intracellular proteins that play a critical role in the regulation of the host innate immune response. Excessive activation of NLRP3 activity is associated with the pathogenesis of various inflammatory diseases; for review, see [86]. These cytosolic receptors are scaffolding proteins that enable assembly of signaling platforms, which trigger signaling pathways (NF-κB and mitogen-activated protein kinase [MAPKs]) and control the activation of inflammatory caspases [87]. Twenty-three NLRs, which have been described in humans [87], have been categorized into five subfamilies according to their N-terminal effector-binding domain: acidic transactivation domain (NLRA); baculovirus inhibitor repeat, BIR (NLRB); caspase recruitment domain, CARD (NLRC); pyrin domain (NLRP); and NLRX1 [88]. NLRs are expressed primarily in phagocytes, including macrophages and neutrophils, although they are also expressed in epithelial cells and astrocytes [87,89].

NLRs sense viral infection by detecting ssRNA [90] and trigger the innate immune response via two pathways; however, the roles of other NLRs during virus infection are also still largely unexplored [90]. The first pathway involves the formation of a multiprotein complex termed inflammasome. This multimeric protein complex consists of NLRP; pro-caspase-1; and occasionally, the adapter apoptosis-associated speck-like protein containing a C-terminal CARD (ASC) [91]. Pro-inflammatory caspase-1 in the inflammasome is processed into active caspase-1, which when released into the cytosol, converts inactive interleukin precursors pro-IL-1β and pro-IL-18 into their mature biologically active forms IL-1β and IL-18, which can then be secreted from infected cells or, in the case of IL-1β, also activate NF-κB (Figure 2) [87,91,92].

NLRP3 is one of the most studied NLRs. NLRP3 inflammasome activation is a tightly regulated process, involving both priming and activation signals. Namely, a pathway leading to activation of NLRP3 inflammasome is initiated by the priming step., i.e., the binding of pathogen-associated molecular patterns (PAMPs) and damage-associated molecular patterns (DAMPs) (e.g., LPS, TNF) to PRRs trigger upregulation of NLRP3, pro-IL-1β, and pro-IL-18 transcription via NF-κB-dependent pathways and posttranslational modifications (PTMs), which appear to be the essential regulators of NLRP3 activation (for reviews, see [86,90,93]). Following priming, PAMPs and DAMPs, such as nigericin, extracellular ATP, and influenza A virus H3N2, act as second activating signal. This signal, which is essential for the formation of the NLRP3 inflammasome, triggers various intracellular events, including K+ efflux, lysosomal disruption, dispersal of the trans-Golgi network, and mitochondrial damage, leading to the release of mitochondrial (mt)DNA and the production of ROS. Priming and activation stimuli jointly induce NLRP3 oligomerization that recruits ASC, triggering the formation of the ASC speck and recruiting pro-caspase-1 (reviewed in [86]). Various RNA viruses can either activate or inhibit NLRP3 inflammasomes (for a recent review, see [94]) via PTMs such as ubiquitination (Ub), phosphorylation (P), sumoylation (S), nitrosylation (N), acetylation (Ace), alkylation (Alk), and ADP-risobylation (ADP) (for a review, see [86]). In addition, the differential DNA methylation pattern of NLRPs may be important for the fate of the cell. Different DNA methylations of NLRP2 between females and males affected SARS-CoV-2 infection and the outcome of COVID-19 disease [95]. Astrocytes have been shown to express NLRP2, NLRP3, and NLRC4 and NLRC5 [89,96,97,98]. Reactive astrocytes upregulate NLRP3 and NLRC4 [97], yet signaling patterns of inflammasome priming and activation in virus infected in astrocytes remain to be revealed.

#### 2.3.3. Retinoic Acid-Inducible Gene I-like Receptors

RLRs are also key sensors of virus infection in the cytosol [99]. The RLR protein family comprises RIG-I, MDA5, and LGP2 receptor [99,100]. RLRs are helicases that sense viral RNA and DNA and, when activated, can trigger signaling cascades, which lead to transcriptional induction of the genes encoding type I IFNs and other immune genes that collectively establish an antiviral host response [99]. Of all the pathogens that infect mammalian cells, RLRs primarily sense viruses [70].

In astrocytes, the constitutive expression of all three RLRs (LPG2, RIG-I, and MDA-5) has been confirmed [101,102]. After virus entry, their expression is upregulated, as demonstrated for LGP2 after infection of mouse astrocytes with negative-sense RNA VSV from the Rhabdoviridae family [101]. Similarly, the expressions of RIG-I and MDA-5 are also increased in mouse astrocytes infected with VSV and another negative-sense RNA virus, Sendai virus, from the Paramyxoviridae family [101]. Both types of viruses also induce robust inflammatory response in astrocytes, as determined by increased release of IL-6 and TNF-α [27]. Finally, in field-RABV-infected human astrocytes, a strong antiviral response is triggered via RIG-I or MDA5 sensing of RABV dsRNA [22].

Similar to TLRs, RLRs are crucial for the production of IFNs; for example, non-enveloped, single-stranded positive-sense RNA TMEV from the Picornaviridae family boosted IFN-β production in astrocytes; due to its immunomodulatory effects, this virus is used as a mouse model for poliomyelitis and multiple sclerosis [63,103].

#### 2.3.4. C-Type Lectin Receptors

CLRs are PRRs expressed at the cell surface that recognize carbohydrate structures on endogenous molecules and pathogens [67,104]. CLRs are predominantly expressed in innate immune cells such as monocytes, macrophages, DCs, and Langerhans cells (LCs) [105]. They comprise two groups: mannose receptor family group I and asialoglycoprotein receptor family group II [106]. CLRs differ from other PRRs with regard to the immediate fate of internalized pathogen. After internalization, a pathogen is degraded either by lysosomes, as demonstrated for DC-SIGN (CD209) and DEC-205 receptors, or via autophagy [105,106]. Regardless of a degradation pathway, antigens are then presented on major histocompatibility complex (MHC) [105,107].

The internalization of viruses via DC-SIGN has been the most studied in DCs, where it has been revealed that, after entry into the host cell, the virus may move along two pathways: the endocytotic pathway, which results in viral degradation and antigen presentation, or the virus is diverted from the endocytotic pathway and avoids degradation [105]. Some viruses target CLRs to evade immune surveillance by suppressing or modulating type I IFNs that play a central role in the innate and adaptive defense against viruses [105]. It is currently unclear how various viruses are engaged in the endocytotic pathway or if they avoid it, and which factors determine the fate of the virus [105]. So far, in astrocytes, only HIV-1 endocytosis has been liked to DC-SIGN receptor [77].

Endocytosed viruses travel via endosomal/lysosomal pathways, where their trafficking is regulated by Ras-related proteins in brain (Rabs) at several levels of the vesicle cycle, for example, at early endosomes (Rabs 4 and 5), at late endosomes and lysosomes (Rab7), and at vesicle-recycling endosomes (Rabs 4 and 11) [6,77,108]. In astrocytes, Rab4, Rab5, and their regulator proteins, guanine nucleotide-dissociated inhibitors (GDIs), crucially affect not only fusion events in endocytosis and recycling but also their molecular interactions with the cytoskeleton, which determine directional vesicle trafficking [109,110,111]. For example, GDI-1 is implicated in all steps of the replication cycle of dengue virus (DENV) in a vascular endothelial cell-like line [112]. Endocytotic trafficking in astrocytes can be diverted on stimulation by IFN, for example, with IFN-γ, which enhances the speed of endosomal and lysosomal vesicles [113]. Such enhanced trafficking could either promote the spread of virus throughout the tissue, thus acting pro-viral, or enhance entry into the degradation pathway in the case of binding to CRLs in astrocytes, resulting in an antiviral outcome. The role of Rabs and their adaptor proteins in viral infections of astrocytes remains to be explored in detail. Moreover, Rabs are also involved in the formation and release of multivesicular body (MVB)-derived exosomal vesicles (EVs) [114,115]. In neurologic disorders, EVs are engaged in transferring various pro-inflammatory or neurotoxic cargos, and they have also been demonstrated to be important for the propagation and release of ZIKV in astrocytes [116,117].

MVB could also function as a virus reservoir in astrocytes, as proposed for HIV-1 in human macrophages [118]. In astrocytes, HIV-1 is predominantly degraded in endosomes, and only a few virions are released intact to spread infection, therefore minutely contributing to the overall viral load in the brain, but may render astrocytes as long-term viral reservoirs [119]. Interconnection between DC-SIGN and MVB in astrocytes certainly deserves further attention. The DC-SIGN receptor binds numerous enveloped viruses via their high-mannose N-linked carbohydrates [120,121]. DI-SIGN was demonstrated to bind DENV, hepatitis C virus, and HIV-1 in human DCs, and WNV in various non-neuronal mammalian cells [106,120,122,123]. In astrocytes, the binding of neurotropic viruses to DC-SIGN, with the exception of HIV-1 [124], has not been explored.

## 3. Viral Infection Triggers Cytokine Signaling and Their Release from Astrocytes

Astrocytes express and release a broad variety of cytokines. Among them are type I IFNs, which are constitutively produced at low quantities in a variety of organs, including the CNS [125,126]. Together with IRFs, IFNs are essential molecules that modulate immune responses after pathogen infection [127]. Astrocytes have high basal expression levels of type I IFNs (IFN-α and IFN-β) together with proteins encoded by ISGs; which allow these cells’ rapid IFN response to restrict viral spread [8]. Astrocytes are the main producers of IFN-β in the CNS after acute infections by various neurotropic viruses and during abortive infection [63]. Such a production of IFN-β was demonstrated during abortive infection with TMEV, VSV, and RABV [63]. Type I IFNs restrict viral growth via autocrine and paracrine signaling through IFNARs, as demonstrated in astrocytes infected with TBEV, JEV, WNV, and ZIKV [8]. Type I IFN mRNA in astrocytes is upregulated quickly on infection, in a matter of hours, and this upregulation has been shown to enhance cell survival [8]. In general, the production of type I IFNs is regulated by the expression of ISG and ISG-linked antiviral activity [128,129]. The principle of type I IFN signaling through autocrine and paracrine binding to IFNARs is conserved and has been extensively reviewed elsewhere [127,128]. Briefly, binding of type I IFNs to IFNARs activates the JAK-STAT signaling pathway, and the transcription of ISG is initiated via a cascade of signaling molecules [128,130]. Antiviral action of ISG encompasses different processes, for example, amplification of IFN signaling, production of cytokines that activate adaptive immunity, direct hindering of virus entry and replication, and degradation of viral RNA and proteins [131].

Although type I IFNs are among the first essential molecules that reduce the virus spread, IFN-γ further assists in virus clearance acting via IFNGR protein complex IFNGR1 and IFNGR2. Both IFNGR complexes initiate the signaling cascade acting on GAS elements in the DNA, thereby inducing cytokine transcription [132] (Figure 2). IFN-γ is secreted predominantly by activated lymphocytes, and natural killer cells, B cells, and professional antigen-presenting cells, including microglia in the CNS [133,134]. IFN-γ stimulates the expression of MHC class II molecules, including in astrocytes, which are transported within late endosomes/lysosomes [113,135]. In astrocytes, MHC class II molecule-transporting vesicles gain speed upon treatment with IFN-γ [113]. This transport is affected by the expression of intermediate filaments (Ifs), glial fibrillary acidic protein (GFAP), and vimentin [113], implying that in reactive astrocytes with boosted IF expression, such traffic is faster. This notion is important from the point of view that, in reactive astrocytes in the neurodegenerative state and during neuroinflammation, the expression of GFAP is increased [136,137]. In addition to GFAP and vimentin, the delivery of MHC class II molecules to the cell surface and fusion of MHC class II vesicles with the plasma membrane are also affected by an increase in [Ca^2+^]_i_ [113]. Enhanced Ca^2+^ signaling in reactive astrocytes has deleterious roles in disease progression [138].

PPRs and IFN-mediated signaling in virus-infected cells evoke the expression of cytokines, including chemokines. Cytokines and chemokines released from virus-infected astrocytes are summarized in Table 2. The differences in chemokines released from human astrocytes compared with mouse astrocytes have been reported, although different developmental stages of astrocytes have not been tested yet in this regard [25].

## 4. Viral Infections of Astrocytes Trigger Neurologic Symptoms

### 4.1. Neurologic Symptoms Induced by Cytokines Released from Astrocytes

On the one hand, IFN-α/β signaling in astrocytes limits early spread of viruses in the CNS [8,149]; on the other hand, cytokines released from astrocytes may act detrimentally to the proper functioning of the CNS. One such harmful effect is closely linked to altered permeability properties of the BBB, where astrocytes together with cerebral microvascular endothelium, pericytes, neurons, and the extracellular matrix form a neurovascular unit [150]. Increased permeability of the BBB on viral infection can be triggered by inflammatory cytokines released from astrocytes and/or by virus-infection-induced downregulation of tight junction (TJ) genes together with the upregulation of metalloproteinases that degrade TJ proteins; these changes have been demonstrated after infections with WNV, TBEV, RABV, and HIV-1 [9,151,152,153,154]. Similar disruptions of TJs are observed in various CNS diseases, such as stroke, multiple sclerosis, cerebral infection, brain tumors, Parkinson disease, and Alzheimer diseases [155,156,157]. Moreover, the dysregulated activation of caspase-1 and IL-1β secretion, as detected in virus-infected astrocytes, is observed in several inflammatory diseases [158]. In addition, NF-κB, which induces the expression of pro-inflammatory genes encoding cytokines and chemokines after viral infection, is also involved in the regulation of CNS neuroinflammation through the regulation of cell survival and the activation and differentiation of innate immune cells [159]. An imbalance in reactive oxygen species, mitochondrial defects, and DNA breakage, which have been previously linked to neurologic disorders, have recently been described after ZIKV infection of induced pluripotent stem cell-derived astrocytes [38]. Along with a deregulation of cellular processes, glial reactivity induced by ZIKV infection has been described in mice and in post-mortem neonatal brain [38].

### 4.2. Effect of Viral Infection on the Glymphatic System and Survival of Neurons

Viral infection of astrocytes may play an important role in the functioning of the glymphatic system. The glymphatic system comprises perivascular tunnels, shaped by astrocytes, which function as a waste clearance system for the elimination of soluble proteins and metabolites from the CNS and facilitate the distribution of glucose, lipids, amino acids, growth factors, and neuromodulators in the brain [160,161]. Proper functioning of the glymphatic system is supported by astrocytic endfeet that surround cerebral endothelial cells in the BBB [160]. One of the hallmarks of astrocyte endfeet is condensed localization of aquaporin-4 (AQP4) water channels that promote the exchange of interstitial and cerebrospinal fluid [160,162,163]. Interestingly, HIV-1 infection instigates a decrease in the expression of AQP4 and its mislocalization, which results in decreased interstitial flow and accumulation of extracellular waste products [160]. This effect is also present after CNS infection with other neurotropic viruses. For example, mislocalization of AQP4 has been demonstrated in an EV71 infection of a mouse brain [164]. On a similar note, DENV infection has been associated with the development of neuromyelitis optica spectrum disorder; the serum of patients tested positive for AQP4 antibody [165]. The pathophysiologic mechanism behind this has not been described yet. Although DENV infection of astrocytes has not been confirmed [166,167], AQP4 antibodies affecting AQP4 localization and function in astrocytes exposes this cell type as a possible contributor to the neurologic symptoms of neuromyelitis optica spectrum disorder.

Virus-infected astrocytes can affect the survival of neurons in contradictory ways. On the one hand, it has been demonstrated that the innate immune response in astrocytes plays a vital role in the injury of dopaminergic neurons, thereby initiating Parkinson disease-like pathology, for example, in cases of infections with WEEV and WNV [19,168,169]. On the other hand, astrocytes, by amplifying the type I IFN response, can limit viral spread in astrocytes and other CNS cells, including neurons, and improve cell survival [8]. A recent study conducted on MHV-A59-infected astrocyte permanent cell lines revealed that astrocytes with various morphology differently produced pro-inflammatory cytokines in response to infection [148]. In reactive astrogliosis, a common consequence of astrocyte infection [139,142,170], astrocytes may adopt multiple phenotypes, which should be defined by a combination of molecular markers and functional readouts [40], and this will be important to take into account in future research on astrocyte infection with viruses that trigger neurologic symptoms.

## 5. Conclusions

To date, a limited number of studies have addressed morphologic and functional changes in astrocytes during progression of neurodegenerative diseases and effects on the neurovascular unit in the CNS, and none of them have focused on viral infections. The involvement of astrocytes in the innate immune response on viral infection is gaining attention as new neurotropic viruses are emerging among the human population and as previously considered non-neurotropic viruses are increasingly being associated with neurologic symptoms. In addition to the immediate consequences of the release of immunomodulatory molecules from astrocytes in the early stages after infection, the implications in chronic inflammation of the CNS tissue during long-term infections also deserves further attention. Moreover, the changes already identified in astrocytes regarding the expression of viral receptors, PPRs, cytokines and their relationship with the surrounding cells need to be revisited from the point of view of direct neurotoxic effects versus indirect immunomodulatory effects and correlated with the accompanying impairments of CNS functions.

## Figures and Tables

**Table 1:** Interactions between pattern recognition receptors (PRRs) and viruses in astrocytes.

Receptor	Virus	Cell/Tissue	Direct and Indirect Effects of Viral Binding to PRRs	References
TLRs (2–4)	polyI:C	human astrocytes	upregulation of TLRs (2–4), secretion of IL-6 and CXCL-10, expression of IFN-β	[73]
TLR7	EV71	cerebral cortex in mice, mouse astrocytes	production of IL-6, apoptosis	[23]
TLR3, TLR4	ZIKV	human astrocytes	increase in the release of RANTES, IP-10, IFN-β, autophagy, TLR3, TLR4 expression	[10]
TLR3	WNV	mouse brain	encephalitis, breakdown of the blood–brain barrier	[74]
RIG-1, MDA-5	VSV, Sendai virus	mouse astrocytes	increase in the expression of RIG-1, MDA-5, release of IL-6, TNF-α	[27]
RIG-1, MDA-5	lab-attenuated RABV	mouse astrocytes	activation of MAVS, production of TNF-α, IFN-γ, IL-6, IL-1β, IL-17, VEGF,	[75]
TLR1-3	lab-attenuated RABV	mouse brain	IFNα/β signaling pathway stimulated expression of many genes encoding inflammatory molecules such as chemokines, cytokines, TLRs (TLR1–3), and complement components	[76]
TLR-dependent MyD88 signaling	TMEV	Mouse astrocytes	release of IFN-β	[63]
DC-SIGN	HIV-1	human astrocytes	Endocytosis of HIV-1	[77]

Table 1 delineates known interactions between pattern-recognition receptors (PRRs) and viruses in astrocytes. Note that interactions between NLRs and viruses are unexplored in astrocytes. poly(inosinic acid):poly(cytidylic acid) (polyI:C); human Enterovirus 71 (EV71); Japanese encephalitis virus (JEV); Zika virus (ZIKV), West Nile virus (WNV); vesicular stomatitis virus (VSV); lab-attenuated rabies virus strain (RABV), Theiler’s murine encephalomyelitis virus (TMEV), mitochondrial antiviral-signaling protein (MAVS), human immunodeficiency virus (HIV-1); myeloid differentiation primary response gene 88 (MyD88).

**Table 2:** Expression of cytokines and interferons in virus-infected astrocytes.

Virus	Cell/Tissue	Inflammatory Cytokines and Chemokines	Chemokines	References
TBEV	primary human astrocytes	TNF α, IFN α, IL-1β, IL-6, IL-8	CCL4/MIP-1β, CXCL10	[9]
WNV	U373 astrocytic cell line	IL-1β	CXCL10, CCL2	[139]
WNV	primary human astrocytes	N/A	CXCL10, CCL5	[140]
ZIKV	primary human brain cortical astrocytes	IL-6, IL-8, IL-12,	CXCL-10, CCL5	[16]
ZIKV	primary human brain cortical astrocytes	IL-6, IL-1α, IL-4, TGF-β1	CXCL-10, CCL5	[10]
JEV	mouse astrocytes (in situ)	N/A	CXCL10	[141]
JEV	primary rat astrocytes	IL-6, TNF-α, IL-1β	CCL5	[142]
JEV	primary human astrocytes	IL-6	CXCL10, CCL2/3/4	[12]
JEV	human fetal astrocyte cell line SVG	IL-18, IL-1β		[143]
SeV	primary mouse astrocytes	IL-6, TNF-α	N/A	[27]
RABV	mouse astrocytes (in situ)	IFN-β	N/A	[63]
SARS-CoV-2	astrocytes differentiated from hiPSCs	N/A ***	N/A	[32]
HSV-1	mouse perivascular astrocytes (in situ) and primary astrocytes		CXCL-1	[144]
HIV-1	primary human astrocytes ****	IL-6, IL-8		[145]
HIV-1	primary human fetal astrocytes	TNF-α, IL-6, IL-8		[146]
VSV	primary mouse astrocytes	IL-6, TNF-α, IFN-β		[27,63]
EV71	mouse brain astrocytes, in situ	IL-6		[23]
TEMV	primary human astrocytes	IL-8	MCP-1	[25]
TEMV	mouse astrocytes (in situ)	IFN-β		[63]
JHMV **	spinal cord astrocytes (in situ)		CXCL10	[147]
MHV-A59 *	astrocyte cell line	IL-1α, IL-1β, IL-2, IL-15, IL-13, IL-17	CXCL10	[148]
TLR3 ligation	human astrocytes	IL-6, IFN-α	CXCL-10	[73]

hiPSCs, human-induced pluripotent stem cells; N/A, not applicable.* Experimental murine coronavirus, murine coronavirus mouse hepatitis virus A-59 (MHV-A59). ** JHM variant V2.2-1 of mouse hepatitis virus (JHMV). *** Not tested in astrocytes, but TLR3/7 and IL-6 were upregulated in neurons. **** Cells transfected with plasmid encoding HIV-1 Nef.

## Data Availability

Not applicable.

## Abbreviations

BBB	Blood–brain barrier
CCHFV	Crimean–Congo hemorrhagic fever virus
CCL2	Monocyte chemoattractant protein-1; MCP-1
CCL4/MIP-1β	C-C motif chemokine ligand 4/Macrophage inflammatory protein 1β
CCL5/RANTES	C-C motif chemokine ligand 5/Regulated upon activation, normal T cell expressed and presumably secreted
CDI	Chronic diffuse infection
CFI	Chronic focal infection
CNS	Central nervous system
CXCL10/IP10	Gamma-interferon-inducible protein-10; IP-10
EBV	Epstein–Barr Virus
EV71	Enterovirus 71
GAS	Gamma interferon activation site
GDI	Guanine nucleotide-dissociated inhibitor
GFAP	Glial fibrillary acidic protein
HCoV-OC43	Human coronavirus OC43
HIV-1	Human immunodeficiency virus 1
HSV-1	Herpes simplex virus-1
IFN	Interferon
IFNAR	IFNα/β receptor
IL	Interleukin
IRF	IFN-regulatory factor
ISG	Interferon-stimulated gene
JEV	Japanese encephalitis virus
MERS-CoV	Middle East respiratory syndrome virus
MHC	Major histocompatibility complex
MVB	Multivesicular body
NLR	NOD-like receptor
PRR	Pattern-recognition receptor
RABV	Rabies virus
SARS-CoV-2	Severe acute respiratory syndrome coronavirus 2
TBEV	Tick-borne encephalitis virus
TGF-β1	Transforming growth factor β1
TI	Tiht junction
TMEV	Theiler’s murine encephalomyelitis virus
VSV	Vesicular stomatitis virus
WEEV	Western equine encephalitis virus
WNV	West Nile virus
ZIKV	Zika virus
